# Supplementary material for: Applications of maximum matching by using bipolar fuzzy incidence graphs
Source: PLoS One. 2023 Aug 22;18(8):e0285603. doi: 10.1371/journal.pone.0285603 (PMC10443885; doi:10.1371/journal.pone.0285603)
Supplement: Attachment — Submitted filename: Response to Reviewer.docx [file pone.0285603.s001.docx]

**Response to Reviewer**

**Title:** Applications of maximum matching by using bipolar fuzzy incidence graphs

**Decision:** Major Revision

Dear reviewers thank you for the encouragement and suggestions. We have revised manuscript carefully according to the given instructions. The below are the comments and reponses of both the reviewers.

**Reviewer # 1: Comments/Suggestion and their responses.**

**Comment 1:Clearly describe the contributions in the abstract section.**

**Reply:** Dear reviewer we are gratified to you for your beneficial comment. We have added our contributions in the abstract. It is in highlighted form in the abstract of revised manuscript.

**Comment 2: An introduction should clearly highlight the motivation, problem statement, the objective of the paper, gap in the existing research and the novelty of the conducted research.**

**Reply:** Thank you for your valuable suggestion. We have improved the introduction section by adding some related material and highlighted it in our revised manuscript. Now, we have added motivation, problem statement, the objective of the paper, gap in the existing research in our revised manuscript in the introductory section on page number 2 in second and third paragraphs. We have highlighted it in our revised manuscript.

**Comment 3: English needs to be improved significantly.**

**Reply:** We are very thankful to you for your valuable suggestion. We have read the whole article carefully and corrected the mistakes and grammatical errors. Also, we have proofread the manuscript thoroughly.

**Comment 4: It would be great if you could improve the comparative analysis section.**

**Reply:**Dear reviewer we have added improved comparative analysis section in the rervised manuscript.

**Comment 5:Expand literature review by citing related work. For example,Graphs for the Analysis of Bipolar Fuzzy Information, Studies in Fuzziness and Soft Computing, Springer, DOI: 10.1007/978-981-15-8756-6, 401(2021).**

**Reply:**Thank you for the suggestion. Now, we have added the above mentioned research work in the reference section at [14].

**Comment 6: min by \min, and max by \max in the whole manuscript.**

**Reply:** We have changed min by \min and \max in our revised manuscript.

**Comment 7:Explain MMBFIN problem in BBFIG more nicely.**

**Reply:**Thank you for the suggestion. Now, we have explained improved MMBFIN problem in BBFIG by discussing also on vertices and added more sections in section 4.

**Reviewer 2: Comments/Suggestions and their responses.**

**Comment 1**:Abstract should be refined. There are some grammar mistakes and

redundant expression which may lead to misunderstandings.

**Reply:** We are gratified to you for your beneficial comment. We have revised the abstract in our revised manuscript and corrected all grammar mistakes and removed the redundant expressions.

**Comment 2: ‘Introduction’ and ‘Preliminaries’ should be shortened. Some of the contents can be merged.**

**Reply:** Thank you for your valuable comment. We have shortened the introduction and preliminaries, with this some contenets are merged now.

**Comment 3: The example used in the paper is considered to illustrate the effectiveness of the proposed approach. However, the authors are suggested to conduct more experiments to show the effectiveness. In addition, more comparisons and analysis should be provided to prove the efficiency.**

**Reply:** Thank you for this suggestion. We have added more subsections in section 4 to illustrate the effectiveness of our proposed approach. Also we have provided more comparison and analysis to show our efficiency.

**Comment 4: Please clearly describe the motivation of this paper in the Introduction Section of this paper.**

**Reply:** Thank you for your valuable suggestion. We have improved the introduction section by adding some related material and highlighted it in our revised manuscript. Now, we have added motivation in our revised manuscript in the introductory section on page number 2 in second and third paragraphs. We have highlighted it in our revised manuscript.

**Comment 5: Please clearly describe the contributions of this paper in the Abstract, the Introduction Section and the Conclusions Section of this paper.**

**Reply:**We are gratified to you for your beneficial comment. We have added our contributions in the abstract, and also added motivation in our revised manuscript in the introductory section on page number 2 in second and third paragraphs. It is in highlighted form in the abstract and introduction section of revised manuscript.

**Comment 6: Please add the following references regarding “Fuzzy graph” into the References Section of this paper and cite them in the Introduction Section of this paper.**

“A Study of m−Polar Neutrosophic Graph with Applications”, Journal of Intelligent & Fuzzy Systems, Journal of Intelligent & Fuzzy Systems, vol. 38, no. 4,pp.4809-4828,2020

“A study on Regular Picture Fuzzy Graph with Applications in Communication Networks” Journal of Intelligent & Fuzzy Systems,2020

**Reply:**Thank you for the suggestion. Now, we have added the above mentioned research work in the reference section at [20] and [21] and cited them in the introduction section.
